# Inhibition of chondroitin sulphate-degrading enzyme Chondroitinase ABC by dextran sulphate

**DOI:** 10.1007/s10719-024-10175-6

**Published:** 2025-01-16

**Authors:** Sagar Dalal, Rachana Pathak, Edward X. S. Moh, Nicolle H. Packer

**Affiliations:** 1 School of Natural Sciences, Faculty of Science and Engineering, Macquarie University, Sydney, NSW 2109 Australia; 2 ARC Industrial Transformation Training Centre for Facilitated Advancement of Australia’s Bioactives (FAAB), Sydney, NSW 2109 Australia; 3 ARC Centre of Excellence in Synthetic Biology, Macquarie University, Sydney, NSW 2109 Australia

**Keywords:** Chondroitinase ABC, Dextran sulfate, Chondroitin sulphate, WST-1, Colorimetric assay

## Abstract

Chondroitin sulphate (CS) is a sulphated glycosaminoglycan (GAG) polysaccharide found on proteoglycans (CSPGs) in extracellular and pericellular matrices. Chondroitinase ABC (CSase ABC) derived from *Proteus vulgaris* is an enzyme that has gained attention for the capacity to cleave chondroitin sulphate (CS) glycosaminoglycans (GAG) from various proteoglycans such as Aggrecan, Neurocan, Decorin etc. The substrate specificity of CSase ABC is well-known for targeting various structural motifs of CS chains and has gained popularity in the field of neuro-regeneration by selective degradation of CS GAG chains. Within this context, our investigation into the biochemistry of CSase ABC led us to a previously unreported inhibition of CSase ABC activity by Dextran Sulphate (DexS). To understand the inhibitory effects of DexS, we compared its inhibition of CSase ABC to that of other polysaccharides such as Heparan Sulphate, Heparin, Colominic Acid, Fucoidan, and Dextran. This analysis identified key structural factors such as monosaccharide composition and linkage, sulphation degree and overall charge as influencing CSase ABC inhibition. Remarkably, DexS emerged as a unique inhibitor of CSase ABC, with distinctive inhibitory effects that correlate with its chain length. DexS has been used to reliably induce ulcerative colitis in mice, effectively mimicking inflammatory bowel diseases in humans, and has been previously shown to inhibit both RNA polymerase and reverse transcriptase. Our investigation emphasizes the interplay between the properties of DexS and CSase ABC, providing significant insights into the utilization of polysaccharide-based inhibitors for modulating enzyme activity.

## Introduction

Chondroitin sulphate (CS) is a sulphated glycosaminoglycan (GAG) polysaccharide commonly found on proteoglycans (CSPGs) in extracellular and pericellular matrices. Its structure consists of long repeating disaccharide units of N-Acetylgalactosamine (GalNAc) and glucuronic acid (GlcA) with a (β−1,3 GalNAc–β−1,4 GlcA)_n_ linkage. CS GAG chains may be sulphated at the 4th and 6th positions of GalNAc or the 2nd position of GlcA or remain unsulphated. The structural complexity and diverse biological activities of CS arise from variations in sulfation degree and patterns across different chain lengths [1]. As a prevalent GAG chain within the extracellular matrix of the central nervous system, CS has been extensively studied for its role as a chemical barrier restricting axonal projections and regeneration [1, 2]. Following spinal cord injury (SCI), the extracellular matrix undergoes alterations, resulting in the development of a glial scar and an upregulation of CSPGs with distinct sulfation patterns [3, 4]. Targeted enzymatic digestion of these chains using Chondroitinases (CSase), a bacterial CS-degrading enzyme, has shown promise in facilitating axonal growth and has been proposed as a potential therapeutic intervention in SCI [5, 6].

**Fig. 1:**
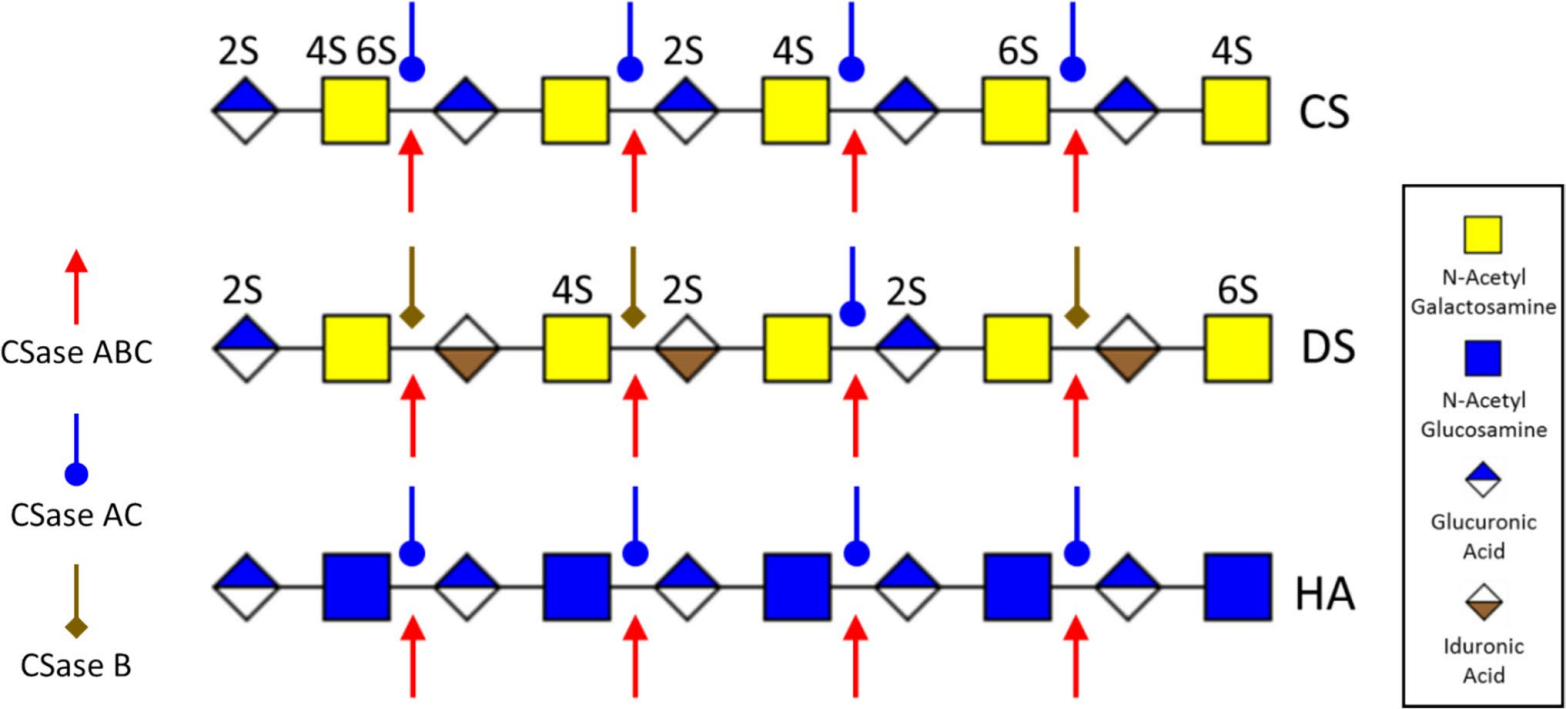
Graphical representation of enzyme cleavage sites for three widely used CSases for digestion of CS, DS and HA GAG chains

The study of the structural diversity of CS chains necessitates the use of microbial enzyme lyases for targeted hydrolysis, yielding disaccharides and tetra saccharides [7]. Hydrolysis of CS using bacterial lyases uses a β-elimination mechanism. This mechanism involves neutralizing the charge of the carboxylic acid group by removing a proton from the chiral center of the glucuronic acid monosaccharide, and liberating the 4-linked hexosamine while creating a C4-C5 double bond within the glucuronic acid ring [8]. Several forms of CSases have been purified from different bacterial strains, but CSase ABC, CSase AC I and CSase B are most used because of their substrate linkage specificities (Fig. 1). Commercially available (Merck Sigma-Aldrich) CSase ABC is a combination of two enzymes purified from *Proteus vulgaris*: CSase ABC I, which has endolytic activity, and CSase ABC II, which has exolytic activity, towards CS, Dermatan Sulphate (DS) and Hyaluronic acid (HA). The repeating disaccharide composition and sulphation pattern of DS is similar to that of CS except some GlcA is epimerized into Iduronic Acid (IdoA). HA is a free non-sulphated GAG chain not attached to a protein core, composing of repeating disaccharide units of N-Acetylglucosamine (GlcNAc) and GlcA. CSase AC from *Arthrobacter aurescens* and *Flavobacterium heparinum* is highly sensitive to the 5-epimerization of GlcA residues in GAG chains and can act on both CS and HA. In contrast, CSase B isolated from *Flavobacterium heparinum* specifically cleaves DS and DS domains in CS-DS hybrid chains [8–10]. This distinct specificity of CSases renders them valuable analytical tools for characterizing CS GAG chains and as potential therapeutic agents in various physiological and pathological conditions such as SCI, glial scars [11] and cancer [12].

While CSase ABC from *Proteus Vulgaris* remains the most extensively studied and characterized enzyme, efforts have been directed towards enhancing its thermal stability and efficiency [13, 14]. However, a gap persists in understanding the regulation of its activity – an attribute which is required if it is to be used therapeutically. Metal cation chelating agents (Zn^2+^, Ni^2+^, Fe^2+^, and Cu^2+^) [15], heparin [16], and short-chain fatty acids have been investigated for their inhibitory effects on CSase ABC [17]. To further investigate the effect of this observed inhibition of CSase by the GAG, heparin, we trialled the potential inhibitory effects of other common, readily available, polysaccharides such as Dextran sulphate (DexS), heparan sulphate, heparin, colominic acid, fucoidan and dextran on the activity of CSase ABC.

## Experimental methods

### Materials and reagents

Chondroitinase ABC (CSase ABC) from *Proteus vulgaris* (C3667 – Sigma Aldrich), CS obtained from shark cartilage (Sigma-C4384) and CS from bovine cartilage (Sigma-C6737). GlcNAc (A8625), Glucose (158968), Dextran Sulphate (D8906), Dextran (D5251), Fucoidan (F8190), Colominic Acid (C5762), Heparan Sulphate (H7640), Heparin (H3149) and WST-1 (5015944001) reagent were obtained from Sigma.

### Measurement of CSase ABC activity

The activity of CSase ABC was measured using a solution containing 5 mU CSase ABC in 20 μl 100 mM ammonium acetate, pH 7, 5 mM CaCl_2_ with 10 μg of CS substrate (5 μg Shark + 5 μg bovine) in a 96 well plate. For inhibition analysis, six different polysaccharides were added to the enzyme–substrate mixture at varying concentrations ranging from 2.5 to 50 μg/ml. Each assay was performed in triplicate with three technical replicates for each condition and statistical analysis was performed using a standard two-tailed t-test to calculate *p*-values.

### WST-1 colorimetric assay for CSase activity

After incubation, reducing end oligosaccharides from digestion of the CS substrate by CSase ABC were quantified, using a modified WST-1 assay [18], to assess the activity of the enzyme. Briefly, a total of 100 μl of WST-1 buffer (5 μl WST-1 (1.69 mM); 95 μl 0.5 M NaOH) was added to each well and incubated at 50 °C for 1 h. Monosaccharide (GalNAc) of known concentrations (0.125 uM—1 mM) were used as standard. Absorbance for each well was measured at 520 nm.

### Length-dependency of DexS

DexS was hydrolysed using 0.1 M Trifluoroacetic acid (TFA) at different time intervals (2 min, 5 min, 15 min, 30 min, 45 min, 60 min) and reconstituted in water after drying under low pressure in a centrifugal evaporator. Hydrolysis was monitored by gel electrophoresis using NUPAGE 4 −12% Bis–Tris polyacrylamide gradient gel (Thermo Fisher Scientific) in MOPS SDS running buffer was performed using 10 ug of hydrolysed DexS from each time point. The gel was extensively washed with water to remove residual SDS, before staining with a cation carbocyanine dye, Stains-all, which has been previously used to visualise charged polysaccharides [19]. Hydrolysed DexS at three concentrations (5, 10, or 20 μg/ml) for all hydrolysis time points was tested for the effect on CSase activity.

### Molecular weight characterization of hydrolysis products

An estimation of the average molecular weight of the DexS hydrolysis products was performed using aqueous gel permeation chromatography (GPC) (1260 II Infinity system with Multi Detector Suite (MDS), Agilent). Acid hydrolysed DexS from each time point was vacuum dried and reconstituted in phosphate buffer saline (PBS, pH 7.2–7.5). Samples were separated on the Advanced BioSEC column (Agilent) at an isocratic flow rate of 0.35 ml/min with a refractive index detector at 30 °C (column and detector) using PBS as a mobile phase. Carbohydrate molecular weight estimation was performed using glucose – 180 Da, and pullulan standards – 6.3 kDa, 47.1 kDa, 642 kDa (Agilent). 250 ug equivalent of DexS was injected for each sample run, and the standards consisted of 50 ug each molecular weight species. The weight-average molecular weight (Mw) of the hydrolyzed DexS products was estimated using Agilent GPC software, which accounts for the molecular weight distribution within the identified chromatographic peaks. Mw was calculated based on methods that are particularly sensitive to variations in molecular size, providing a more accurate representation of the molecular weight distribution of the hydrolyzed DexS.

## Results and discussion

### Dextran sulphate inhibits the activity of CSase ABC

**Fig. 2:**
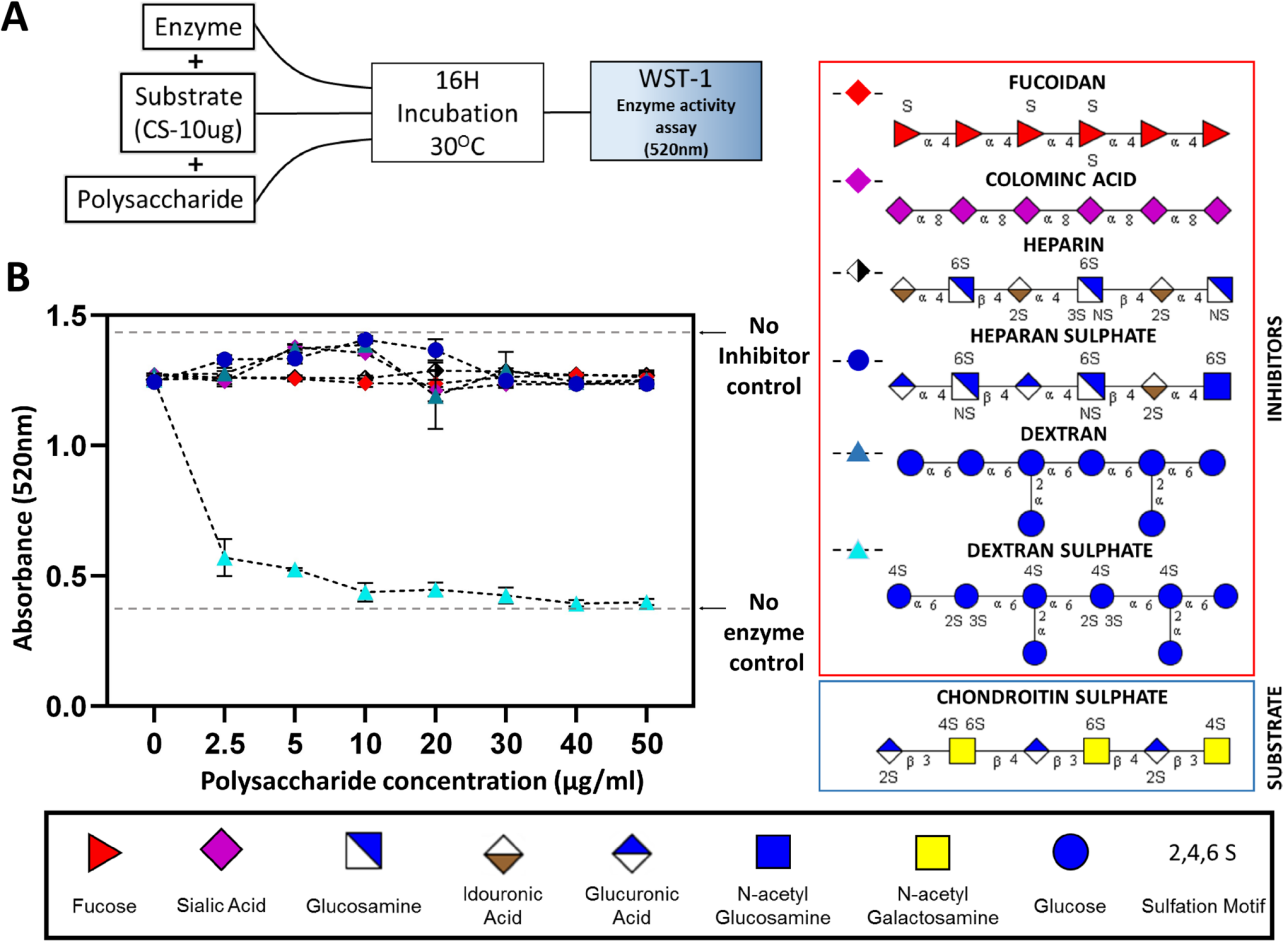
**(A)** Experimental workflow to measure CSase ABC activity with CS substrate (10 ug) when incubated with Fucoidan (200 KDa), Colominic Acid (150 KDa), Heparin (15–19 KDa), Heparan Sulphate (120–140 KDa), Dextran (500 KDa) and Dextran Sulphate (500 KDa) at increasing concentrations. (**B**) Inhibitory effects of different polysaccharides measured using WST-1 colorimetric assay that measures reducing termini of the hydrolysed CS substrate

Inhibition of CSase activity against CS substrates was assessed using a colorimetric assay with WST-1 dye (Fig. 2A) which undergoes a reductive reaction, resulting in the formation of coloured formazan in the presence of a reducing end [18, 20, 21]. A positive correlation between absorbance and CS hydrolysis is a measure of enzyme activity, with higher absorbance indicating increased activity. When the inhibitory effects of polysaccharides of varying compositions, degrees of sulfation, linkages/branching, and charges (Fig. 2B) were compared, surprisingly DexS was the only inhibitor of CSase ABC activity (Fig. 2C), reaching maximum inhibition at a concentration of 10 μg/ml (40 pmol). Interestingly, even at a low concentration of 2.5 μg/ml, DexS significantly reduced enzyme activity, approaching levels comparable to the control with no enzyme added.

Importantly, the non-sulphated form of DexS, Dextran, showed no inhibition of CSase ABC. Martinez et al. has previously showed approximately 81% inhibition of CSase ABC using heparin with a 1:1 substrate to inhibitor ratio. Significantly, the potency of DexS is substantially greater, achieving close to 90% inhibition with a 10:1 substrate to inhibitor ratio against CSase ABC. Other highly negatively charged polysaccharides, such as fucoidan (sulphate groups) and colominic acid (carboxylic acid groups), did not inhibit CSase ABC activity at these concentrations. DexS has been previously identified as a potent inhibitor of other GAG lyases, such as hyaluronidase where DexS was found to be a non-toxic inhibitor of HA degradation observed in a human breast adenocarcinoma cell line [22]. DexS has also shown promise in inhibition of HIV-1 and other viral infections [23].

### Length-dependency of DexS on CSase ABC inhibition

**Fig. 3:**
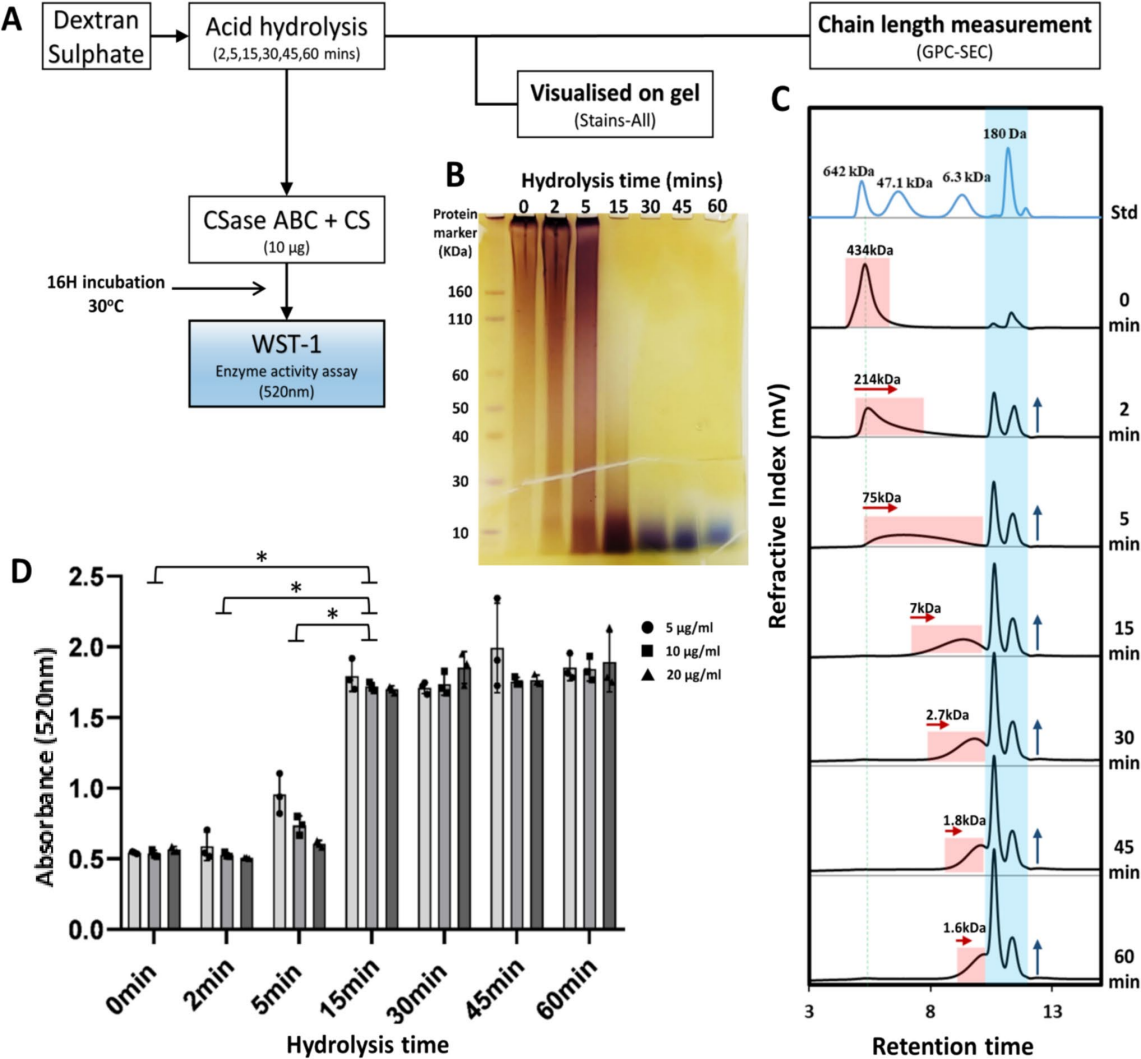
**(A)** Experimental workflow to test for length dependency of DexS on inhibition of CSase activity. (**B**) SDS-PAGE showing reduced chain length of DexS with increase in acid hydrolysis time. (**C)** GPC-SEC chromatogram estimating the molecular weight of hydrolysed DexS products at each time point, with the red box indicating a decrease in polymer chain length and the blue box highlighting the increased mono- and disaccharide products. (**D)** Inhibition of CSase ABC activity at different DexS hydrolysis times was tested using three concentrations of DexS. Enzyme activity was assessed by the WST-1 assay, performed in triplicate. A standard two-tailed t-test was used to calculate p-values, with "*" indicating *p < *0.01

To investigate whether the length of the DexS polymer influences its inhibition of CSase ABC activity, CSase ABC activity were evaluated against a range of acid hydrolysed DexS products (Fig. 3A). Acid hydrolysis of DexS was validated via SDS-PAGE, visualized with Stains-All (Fig. 3B), showed a progressive reduction in DexS chain length with increasing hydrolysis time. The molecular weight of the hydrolysed DexS products was estimated by GPC, revealing a decrease in DexS size (Fig. 3C), consistent with the SDS-PAGE gel (Fig. 3B). The pullulan standard used in the GPC is a mix of linear polysaccharides, and was used for the estimation of the molecular weights of the hydrolysed DexS.

The inhibitory effects of the decreasing DexS polymer chain length on CSase ABC activity were assessed at identified inhibitory concentrations of DexS (5, 10, and 20 μg/ml) (Fig. 2C). Notably, DexS exhibited a diminished inhibitory effect as its chain length decreased (Fig. 3D). After 15 min of hydrolysis, the shorter, lower molecular weight, chains showed no inhibition of CSase activity. A comparison between the 2-min and 5-min hydrolysis time points with the 15-min time point revealed a clear size-dependent inhibitory effect, with DexS polymers of 7 kDa or lower exhibiting no inhibition of CSase ABC activity.

Changes in the chain length of DexS have previously been demonstrated to impact its activity as both an anti-coagulant and an inhibitor of complement pathways. In immunology, lower molecular weight DexS (LMW DexS, < 10 kDa) has shown efficacy in inhibiting all three complement pathways, a key mechanism in pathogen recognition in innate immunity [24–26]. LMW DexS has also been utilized as an endothelial cell protectant against NK cell-mediated cytotoxicity, highlighting its potential to mitigate proinflammatory effects of innate immune mediators [27]. Further emphasising the chain length dependency of DexS activity, recent research compared different sizes of DexS as an antiviral agent against SARS-CoV-2 [28]. With no observed toxic effects in Vero cells, 500 kDa DexS exhibited robust antiviral properties compared to shorter chains, demonstrating similar antiviral efficacy to the clinically approved drug Remdesivir. These findings also suggested that long chain DexS intervention primarily targets the early stages of infection, positioning it as a promising candidate for antiviral therapy [28, 29]. However, challenges remain in optimizing DexS administration to enhance its bioavailability, contributing to current skepticism regarding the use of polymer-based viral inhibitors [29].

Our study describes the inhibition of CSase ABC activity specifically by DexS polymers and that this inhibition is dependent on monosaccharide type, sulphation and polymer chain length. Dextran Sulphate (DexS), a sulphated analog of dextran is a highly water-soluble polymer previously known for its inhibitory effects on the activity of RNA polymerase and reverse transcriptase [30] and of bovine testicular hyaluronidase (HAase) and bacterial HAase [22]. In this study, we provide insights into the use of DexS for modulating bacterial CSase ABC and highlight the impact of varying DexS lengths on its inhibitory effects.

Further evaluation of other known homologues of CS lyases and other GAG-degrading enzymes will be interesting to determine in the future. Approaching CSase ABC from a biochemical perspective, this study may offer a mechanistic insight into enzyme inhibition by a single polymer, DexS, which has not previously been known to inhibit CS degradation. In addition, this work provides a platform to evaluate polysaccharide inhibitors of GAG-specific enzymes. It broadens the scope of enzyme assays to include a wider range of GAG-degrading enzymes and supports enzyme inhibition research using GAG-mimetic and other small molecules. This assay could be a potential tool for quality control in inhibitor-based therapeutics, aiding in the identification and optimization of effective inhibitors.
